# Laterally Placed Expandable Interbody Spacers With and Without Adjustable Lordosis Improve Radiographic and Clinical Outcomes: A Two-Year Follow-Up Study

**DOI:** 10.7759/cureus.20302

**Published:** 2021-12-09

**Authors:** Yan M Li, Zheng Huang, James Towner, Yan I Li, Brandon S Bucklen

**Affiliations:** 1 Neurosurgery and Oncology, University of Rochester Medical Center, Rochester, USA; 2 Orthopaedics, Guanghua Hospital, Shanghai, CHN; 3 Research, Globus Medical Inc., Audubon, USA

**Keywords:** expandable interbody spacers, adjustable lordosis, mis llif, degenerative disc disease, sagittal correction

## Abstract

Introduction

Interbody spacers are necessary for achieving disc height restoration when surgical intervention is used for the treatment of severe degenerative disc disease. Minimally invasive lateral lumbar interbody fusion (MIS LLIF) is a popular surgical approach that historically uses large static interbody spacers through a lateral approach. However, static spacers have been associated with iatrogenic distraction and excessive impaction forces, which may increase the risk of subsidence and loss of lordosis, compromising stability. Expandable interbody spacers with or without adjustable lordosis may help address these concerns by maximizing segmental lordosis and aiding in sagittal balance correction. This study describes the clinical and radiographic outcomes of patients treated with expandable interbody spacers with or without adjustable lordosis, for MIS LLIF.

Materials and methods

This is retrospective, single-surgeon Institutional Review Board-exempt chart review was of 103 consecutive patients who had undergone MIS LLIF at one to two contiguous level(s) utilizing expandable interbody spacers with or without adjustable lordosis (66/103 patients had adjustable lordosis spacers). Collection of clinical and radiographic functional outcomes occurred at preoperative and postoperative time points through 24 months.

Results

One-hundred and three consecutive patients were evaluated-average age, 58.2 ± 12.1 years; 42.1% (45/107) were female. There were 78.6% (81/103) one-level cases and 21.4% (22/103) two-level cases for a total of 125 levels; 44.8% (56/125) were performed at L4-5 and 34.4% (43/125) at L3-4. The average estimated blood loss was 24.6 ± 12.3cc. Mean operative time was 61.0 ± 19.1 min, and mean fluoroscopic time was 28.2 ± 14.6 sec. Visual Analog Scale (VAS) back and leg pain scores decreased significantly by an average of 7.1 ± 1.0 points at 24 months (*p*<0.001). Oswestry Disability Index (ODI) scores significantly decreased by a mean of 67.4 ± 8.9 points at 24 months (*p*<0.001). Lumbar lordosis significantly improved by a mean of 3.1 ± 8.8° at 24 months (*p*=0.001). Anterior, middle, and posterior disc height significantly increased at 24 months by averages of 4.7 ± 3.1, 4.0 ± 3.0, and 2.1 ± 2.2mm, respectively (*p*<0.001). Neuroforaminal height had significantly increased at 24 months by a mean of 3.0 ± 3.6mm (*p*<0.001). Segmental lordosis significantly improved by 3.7 ± 2.9° at 24 months (*p*<0.001). There were 51 patients with abnormal preoperative Pelvic Incidence-Lumbar Lordosis (PI-LL) measurements that significantly improved by 9.1 ± 4.9° (*p<*0.001) and 52 patients with normal preoperative PI-LL measurements that improved by 0.2 ± 4.6° (*p=*0.748) at 24 months. One-hundred percent fusion occurred at all levels, and no findings of radiolucency were observed. One case of subsidence (1/125, 0.8%) was reported at 24 months. No implanted-related complications were reported, with 0% pseudoarthrosis and no secondary surgery required at the operative levels.

Conclusion

Indirect decompression and sagittal correction were achieved and maintained through a 24-month follow-up. Functional clinical outcomes significantly improved based on decreased VAS pain and ODI scores at 24 months. This study resulted in positive clinical and radiographic outcomes for patients who underwent MIS LLIF with expandable interbody spacers with or without adjustable lordosis.

## Introduction

A significant contributing factor for low back pain is the progressive irreversible condition known as degenerative lumbar disc disease (DDD), the leading cause of chronic back pain for the aging population. Life-altering disability may result from associated radiculopathy, spinal stenosis, myelopathy, degenerative spondylolisthesis, and herniation manifested from loss of disc height and lordosis [1]. When conservative treatments fail, lumbar interbody fusion is an effective surgical option to relieve pain and restore quality of life [2]. Restoration of alignment and stabilization until fusion occurs is the goal of spinal arthrodesis and is associated with improved clinical outcomes [3]. There are a variety of surgical techniques that allow access to the disc space for lumbar interbody fusion {posterior lumbar interbody fusion (PLIF), transforaminal lumbar interbody fusion (TLIF), oblique lumbar interbody fusion (OLIF), anterior lumbar interbody fusion (ALIF), and lateral lumbar interbody fusion (LLIF)}. Each has its own advantages and disadvantages with several anatomical trajectories. Potential trade-offs are determined by anatomic obstacles, feasibility of graft placement, degree of local supportive structure resection, and optimization of graft size [4-8].

In 2006, Ozgur first described LLIF as extreme lateral interbody fusion (XLIF) and since then, the lateral approach has gained popularity [6]. This approach allows access to the lumbar region through a lateral approach, passing through the psoas major and retroperitoneal space. The lateral approach is an excellent option for treating lumbar degenerative diseases by achieving decompression with smaller incisions, lower estimated blood loss, and shorter length of hospital stay after surgery [5-7]. Achievement and maintenance of sagittal alignment is a significant predictor of clinical outcomes and may minimize adjacent segment degeneration [3]. Postoperative sagittal abnormalities such as a hypolordotic alignment may result in adjacent segment disease (ASD) and poor clinical outcomes. Therefore, to achieve sufficient indirect decompression, it is essential to maximize disc and neuroforaminal height while simultaneously maintaining lumbar and segmental lordosis [9-11].

Promising clinical outcomes on the LLIF approach as a reliable approach to achieving the aforementioned surgical goals have been reported. LLIF permits the use of large spacers while preserving primary stabilizing structures that aid in stability and sagittal correction [8]. Due to reports of muscle-sparing dissection, shorter operative, and recovery time, reduced postoperative pain, direct visualization of the disc space allowing for optimal disc preparation, spacer size, and placement, and decreased soft tissue retraction, minimally invasive lateral lumbar interbody fusion (MIS LLIF) has gained popularity [5].

To achieve optimal decompression and sagittal correction, large static interbody spacers have traditionally been the standard of care for lumbar fusion [2]. However, static interbody spacers have been associated with iatrogenic distraction during interbody insertion, increasing the chance of subsidence leading to a loss of disc height and lordosis, which may compromise stability [12-14]. These concerns can be addressed through the use of an expandable lateral interbody spacer, in conjunction with the LLIF approach, allowing for controlled restoration of disc height and diminished impaction forces, thereby providing indirect decompression and maximizing patient outcomes through the muscle-sparing lateral approach [15,16]. As with any new technology, clinical outcome studies are needed to determine the clinical utility of these devices. The goal of this study is to evaluate the radiographic and clinical outcomes of patients who underwent MIS LLIF using expandable interbody spacers with and without adjustable lordosis through two years postoperative.

## Materials and methods

Patient population

**Figure 1:**
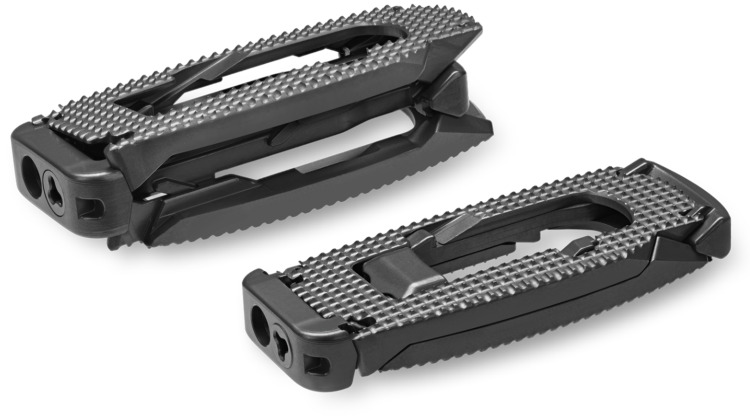
Expandable interbody spacer with adjustable lordosis (RISE®-AL, Globus Medical, Inc., Audubon, Pennsylvania, USA) in expanded and minimized forms

**Figure 2:**
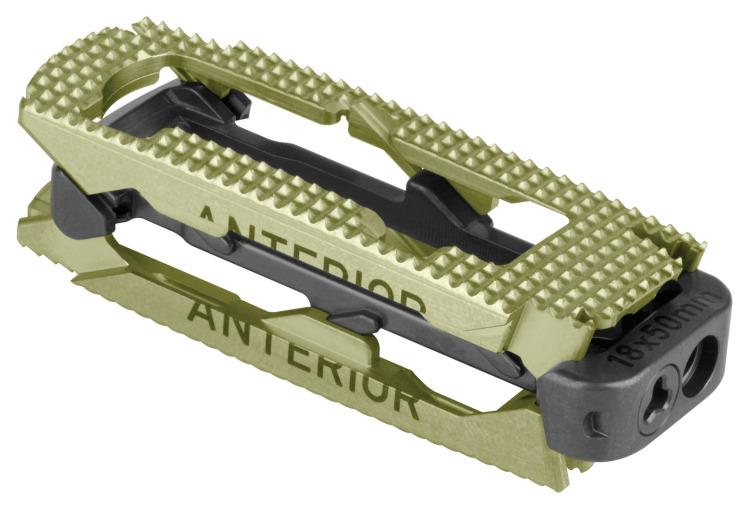
Expandable interbody spacer (RISE®-L, Globus Medical, Inc., Audubon, Pennsylvania, USA)

This is a retrospective, single-surgeon, Institutional Review Board (The University of Rochester Office for Human Subject Protection)-exempt evaluation of a prospectively collected cohort of 103 patients. The patient population was diagnosed with DDD at one or two contiguous levels from L1 to L5 with or without Grade 1 spondylolisthesis. Some were also diagnosed with spinal stenosis (30) or degenerative spondylolisthesis (3). All patients underwent an MIS LLIF surgery using a novel titanium expandable interbody spacer with (RISE®-AL, Globus Medical, Inc., Audubon, Pennsylvania, United States) (Figure 1) or without (RISE®-L, Globus Medical, Inc.) (Figure 2) adjustable lordosis, with supplemental posterior screw and rod fixation (Figures 1-2), from August 2016 to January 2017. Patients were included in the analysis if they were 18 years of age or older but less than 80 years, underwent a one- and/or two-level surgery, had no history of previous fusion attempted at the operative level, were not diagnosed with a condition that would interfere with bony fusion/healing, had no history of alcohol and/or drug abuse, and smoked less than one pack per day. All patients who smoked were required to quit smoking two to three weeks prior to surgery and have a negative nicotine test. Spinal stenosis with neurogenic claudication and degenerative spondylolisthesis were the most common diagnoses. A prospectively collected database using radiographic records and patient self-assessment forms was used to assess clinical and radiologic outcomes. Data were collected preoperatively and at six weeks, three, six, 12, and 24 months postoperatively.

Surgical technique

After administrating general anesthesia, patients were put in the lateral decubitus position and secured by adhesive tape to a radiolucent table. The break was positioned at the greater trochanter with the iliac crest above it. With fluoroscopic guidance, an oblique incision was made at the symptomatic disc segment allowing for direct visualization through subcutaneous tissue, external and internal oblique muscles, and transversus abdominis. A blunt dissection was performed and retroperitoneal fat was mobilized anteriorly; this exposed underlying psoas muscle, which was then palpated. After fluoroscopic confirmation of the level and location of the spinal marker, another blunt dissection was performed anteriorly; this was done to or at the very anterior part of the psoas muscle to the operative intervertebral disc level.

**Figure 3:**
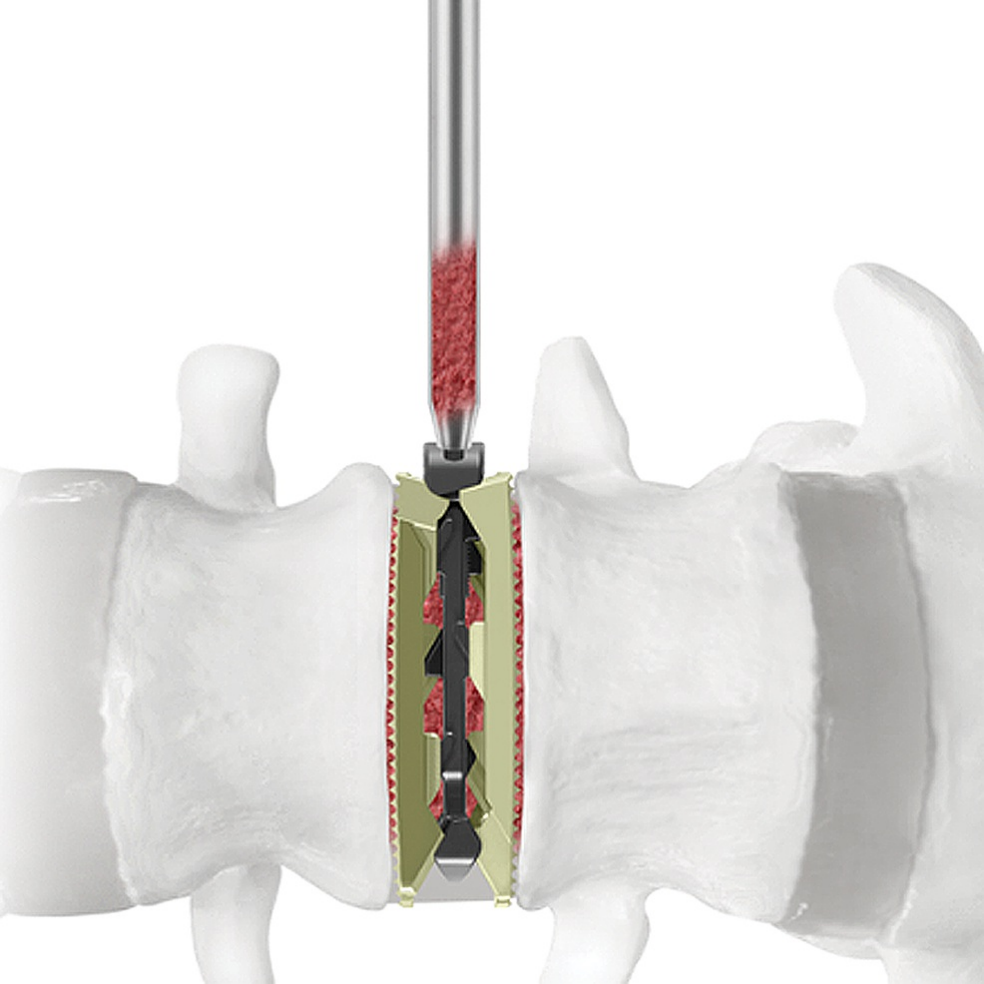
Implant graft chamber Additional autogenous bone graft may be packed into the graft chamber of the implant after expansion and around the implant if desired.

Neuromonitoring stimulation was done in the initial step and whenever retractors were relocated. After the appropriate level was confirmed by fluoroscopy, a minimally invasive retractor was docked, dilated at the segment, and secured to the table-mounted arm. An annulotomy was next performed, which was followed by discectomy and endplate preparation. Using fluoroscopic imaging, sufficient endplate preparation was then completed and confirmed, allowing for trials to be inserted into the disc space for gradual distraction and determination of the appropriate spacer size for the patient. Once the appropriate expandable interbody spacer was selected and packed with local autograft, it was then implanted at the middle or slightly anterior middle of the disc space (Figure 3). The spacer was inserted at a contracted height followed by expansion of the spacer in situ to the desired height once correctly positioned within the intervertebral disc space and backfilled with local autograft bone (Figure 3). The desired expanded height was confirmed by both fluoroscopy and tactile feel of the implant with the driver and counting the number of revolutions of the driver to expand the implant. One revolution is equivalent to 0.5mm of expansion. Expansion in situ allows for endplate-to-endplate contact and, for the AL (lordotically-actuated) spacer, adjustable lordosis.

Posterior decompression was performed for cases of severe spinal stenosis with neurological deficits, or in cases in which preoperative disc height had not increased by over 100% due to the LLIF procedure. Pedicle rods and screws were utilized for supplemental fixation. When the rods were properly positioned, locking caps were set. Intraoperative fluoroscopy images were taken of the final construct for confirmation and final approval, followed by cleaning and closure of the surgical incisions in the standard fashion. Antibiotic prophylaxis was administered to prevent infection.

The two lateral interbody spacers used in the current study were height-actuated (RISE^®^-L) and lordotically actuated (RISE^®^-AL) spacers. RISE^®^-L and RISE^®^-AL spacers are expandable LLIF devices manufactured from titanium alloy, designed to maximize indirect decompression, provide a large graft space for optimal fusion, and minimize impaction forces. These devices are inserted at a contracted height and expanded up to 7mm in situ. The footprint options include 18 and 22mm widths, five lengths (40-60mm, in 5mm increments), 7-17mm height (height range dependent on lordosis), and in parallel or lordotic profiles. The height-actuated spacer is a fixed lordotic endplate of either 6° or 10°. The lordotically actuated spacer allows up to 3-15° of adjustable lordosis. The 18mm-wide implant was most commonly used in this study at 90%, versus the 22mm.

For prophylaxis, three doses (weight dependent, 1-2 grams) of antibiotics were administered at preoperative, intraoperative, and immediate postoperative time points.

Outcome measures

Fluoroscopy and operative times were recorded during LLIF procedures. Primary outcomes included self-assessment patient questionnaires such as Visual Analog Scale (VAS) for back and leg pain and Oswestry Disability Index (ODI), which were evaluated preoperatively and at six weeks, three, six, 12, and 24 months postoperatively.

**Figure 4:**
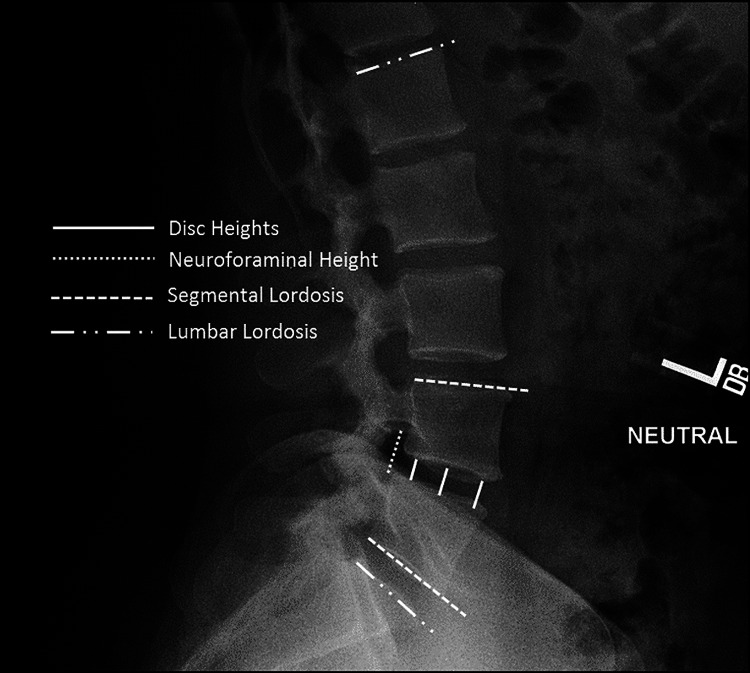
Standing lateral lumbar spine radiograph with superimposed lines displaying the measurements evaluated in this study Measurements included disc heights, neuroforaminal height, segmental lordosis, and lumbar lordosis

Radiographic assessments were also considered primary outcomes. Radiographic parameters such as disc height (disc space anterior, middle, and posterior locations), segmental lordosis, neuroforaminal height, Pelvic Incidence-Lumbar Lordosis (PI-LL), and lumbar lordosis were evaluated at six weeks, three, six, 12, and 24 months. Radiographic lumbosacral parameters were measured on upright standing, lateral radiographs using imaging software (Surgimap®, Globus Medical Inc., Audubon, Pennsylvania, United States; Intellispace PACS 4.4, Koninklijke Philips N.V., Amsterdam, Netherlands) (Figure 4), by a trained researcher and verified by an orthopaedic surgeon, just above and below referenced index levels on the lateral plane.

Secondary outcome measures of demographic, perioperative, and radiographic data were recorded from preoperative to two years postoperative. Segmental lordosis was measured from the inferior endplate of the caudal vertebral body to the superior endplate of the cephalad vertebral body. Neuroforaminal height was measured as the distance from the inferior pedicle wall of the level above to the superior pedicle wall of the level below. Lumbar lordosis was measured from the endplate of S1 to the L1 superior endplate (Figure 4).

Intervertebral fusion, postoperative surgical and hardware-related adverse events (implant subsidence, breakage, and expulsion), radiolucency, ASD, and pseudoarthrosis were reported at 24-month follow-up for each patient. ASD was assessed clinically and in correlation with radiographic studies. Two independent observers assessed fusion with an agreed consensus using flexion/extension x-rays at two-year follow-up and cross-referenced by Fogel et al. for fusion grading following the Brantigan, Steffee, and Fraser (BSF) radiographic classification criteria [17]. According to this classification, BSF-1 is radiographic pseudoarthrosis, BSF-2 is radiographical lock pseudoarthrosis, and BSF-3 is radiographical fusion (Table 1). Subsidence was defined as a measured reduction in disc height greater than 3mm at 24 months postoperatively compared to disc height at six weeks postoperatively [18,19].

**Table 1:** Classification of interbody fusion success: Brantigan, Steffee, Fraser (BSF)

Classification of interbody fusion success: Brantigan, Steffee, Fraser (BSF)
BSF-1: Radiographical Pseudarthrosis is indicated by a collapse of the construct, loss of disc height, vertebral slip, broken screws, displacement of the carbon cage, or significant resorption of the bone graft, or lucency visible around the periphery of the graft or cage.
BSF-2: Radiographical Lock Pseudarthrosis is indicated by lucency visible in the middle of the cages with solid bone growing into the cage from each vertebral endplate.
BSF-3: Radiographical Fusion: bone bridges at least half of the fusion area with at least the density originally achieved at surgery. Radiographical fusion through one cage (half of the fusion area) is considered to be mechanically solid fusion even if there is lucency on the opposite side.

Statistical analysis

IBM SPSS Statistics for Windows, Version 20.0 (Released 2011; IBM Corp., Armonk, New York, United States) was used for statistical analysis. Frequency analyses and percentages were used for descriptive statistics. Paired sampled t-tests were used to calculate significant changes in ordinal and interval variables from preoperative to each postoperative follow-up time. Independent t-tests were used to determine significant differences between expandable groups, and between one- and two-level procedures. Statistical significance was found at p<0.05.

## Results

Patient demographic and operative data

From August 2016 to November 2017, 103 consecutive patients underwent MIS LLIF, with 66/103 (64.1%) patients implanted with an expandable interbody spacer with adjustable lordosis (AL) and 37/103 (35.9%) patients implanted with an expandable interbody spacer without adjustable lordosis (L). There was an average age of 58.2 ± 12.1 years and 43.7% (45/103) were female (Table 2).

**Table 2:** Baseline characteristics AL: expandable interbody spacer with adjustable lordosis; L: expandable interbody spacer without adjustable lordosis

Baseline Characteristics			
Parameter	AL	L	Overall
Number of Patients	66	37	103
Sex			
Female, n (%)	31 (30.1%)	14 (13.6%)	45 (43.6%)
Male, n (%)	35 (34.0%)	23 (22.3%)	58 (56.3%)
Age, mean (SD, range)	58.0 (12.1, 21–82)	60.3 (12.0, 34–82)	57.8 (12.6) (21–76)

Surgical data

Of the 103 patients, 78.6% (81/103) were one-level (1L) cases (53 AL patients, 28 L patients) and 21.4% (22/103) underwent a two-level (2L) MIS LLIF (13 AL patients, nine L patients), for a total of 125 treated spinal fusion levels (79 total AL levels, 46 total L levels). Of the 125 levels, 44.8% (56/125) occurred at L4-L5, and 34.4% (43/125) occurred at L3-L4. Neuromonitoring did not show any nerve conduction abnormalities (lumbar plexus) or signal changes. Mean operative time was 54.4 ± 12.6 min for 1L fusions and 85.5 ± 19.2 min for 2L fusions. Mean fluoroscopic times were 27.4 ± 13.6 sec for 1L fusions and 30.9 ± 17.8 sec for 2L fusions. Length of hospital stay was 3.4 ± 1.9 days for 1L fusions and 4.4 ± 2.6 days for 2L fusions. Mean estimated blood loss for 1L fusions was 23.6 ± 13.2 cc and for 2L fusions 28.0 ± 7.7 cc (Table 3).

**Table 3:** MIS LLIF fusion procedure characteristics AL: expandable interbody spacer with adjustable lordosis; L: expandable interbody spacer without adjustable lordosis

Parameter	AL	L	Overall
Type of Surgery, n (%)			
One-level	53 (51.5%)	28 (27.2%)	81 (78.6%)
Two-level	13 (12.6%)	9 (8.7%)	22 (21.4%)
Levels Treated, n (%)			
L1-L2	4 (3.2%)	1 (0.8%)	4 (4.0%)
L2-L3	11 (8.8%)	10 (8%)	22 (17.6%)
L3-L4	26 (20.8%)	17 (13.6%)	43 (34.4%)
L4-L5	38 (30.4%)	18 (14.4%)	56 (44.8%)
Mean Estimated Blood Loss, n (SD)			
One-level	24.7 (13.6)	21.7 (12.3)	23.6 (13.2)
Two-level	30.8 (7.3)	23.9 (6.5)	28.0 (7.7)
Mean Operative Time, n (SD)			
One-level	52.6 (10.7)	57.8 (15.3)	54.4 (12.6)
Two-level	79.9 (20.8)	93.6 (14.0)	85.5 (19.2)
Mean Flouroscopic Time, n (SD)			
One-level	29.8 (14.3)	23.0 (10.9)	27.4 (13.6)
Two-level	29.8 (13.5)	32.4 (23.4)	30.9 (17.8)
Mean Length of Hospital Stay, n (SD)			
One-level	3.1 (2.0)	3.8 (1.6)	3.4 (1.9)
Two-level	4.5 (2.8)	4.2 (2.2)	4.4 (2.6)

Few minor perioperative complications were reported, with a 10% ileus complication rate, which resolved a few days after surgery, and 10-15% thigh numbness, which resolved on its own by two to six weeks postoperatively. There were no reported cases of surgical infection for the patient population. Moreover, no cases of vascular, bowel, or plexus injuries were observed.

Clinical outcomes

**Figure 5:**
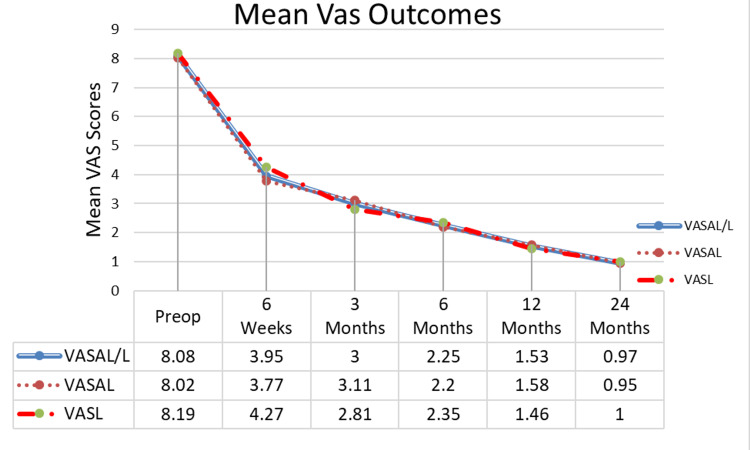
Mean VAS back pain The results show a significant decrease in VAS back pain scores from baseline and sustained at 1.5, 3, 6, 12, and 24 months. VALAL/L- are the mean VAS scores for AL and L patients combined (103 patients); VASAL-AL patients only (66 patients); VASL-L patients only (37 patients) VAS: Visual Analog Scale; AL: expandable interbody spacer with adjustable lordosis; L: expandable interbody spacer without adjustable lordosis

**Figure 6:**
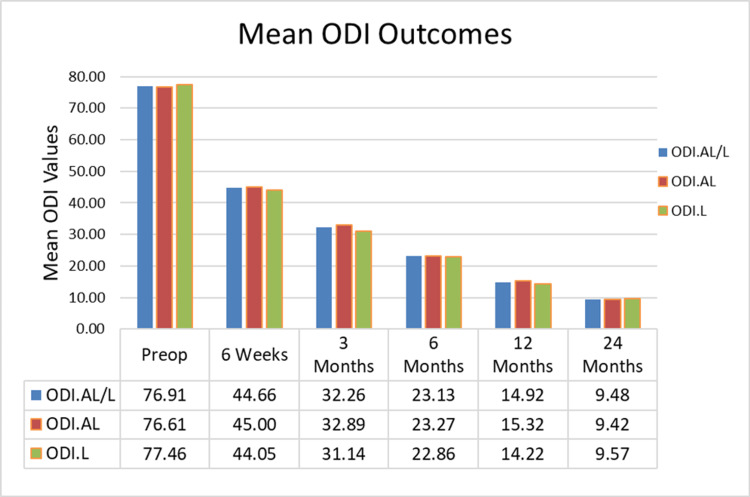
Mean ODI scores The results show a significant decrease in ODI back scores from baseline and sustained at 1.5, 3, 6, 12, and 24 months. ODI.AL/L- mean scores for AL and L patients combined (103), ODI.AL- AL patients only (66), and ODI.L- L patients only (37) ODI: Oswestry Disability Index; AL: expandable interbody spacer with adjustable lordosis; L: expandable interbody spacer without adjustable lordosis

Patients reported significant improvements in pain and disability at final follow-up from baseline. Mean VAS scores significantly decreased at six weeks, three, six, 12, and 24 months postoperatively by 51.1% (4.1 ± 1.1), 62.9% (5.1 ±1.2), 72.2% (5.8 ± 1.3), 81.1% (6.5 ± 1.3), and 88.0% (7.1 ± 1.0) points, respectively (p<0.001) (Figure 5). Mean ODI scores improved significantly from baseline by an average of 41.9% (32.3 ± 13.1), 58.1% (44.7 ± 12.1), 69.9% (53.8 ± 13.1), 80.6% (62.0 ± 12.4), and 87.7% (67.4 ± 8.9) points at six weeks and three, six, 12, and 24 months, respectively (p<0.001) (Figure 6).

Radiographic outcomes

**Figure 7:**
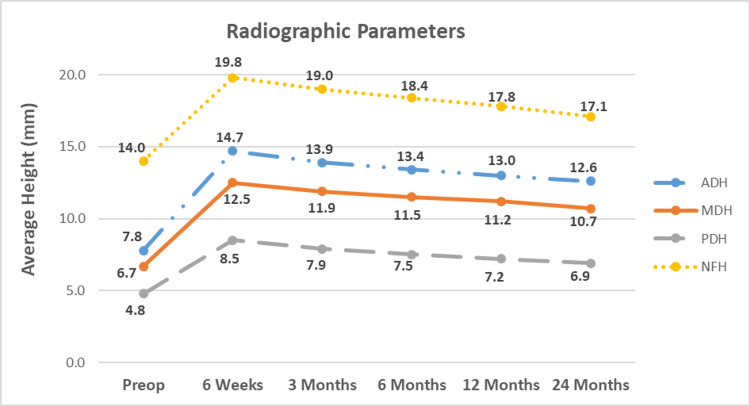
Average anterior, middle, posterior disc heights and neuroforaminal heights Significant improvement from baseline (preop) values were achieved at 6 weeks, 3, 6, and 12 months postoperatively, and sustained at 24-month follow-up. ADH: anterior disc height; MDH: middle disc height; PDH: posterior disc heights; NFH: neuroforaminal height

Lumbar lordosis significantly improved at six weeks and three, six, and 12 months and was sustained at 24 months by 16.9% (6.8 ± 8.3°), 14.4% (5.8 ± 7.9°), 12.0% (4.9 ± 8.5°), 10.7% (4.3 ± 8.6°), and 7.6% (3.1 ± 8.8°), respectively (high standard deviation due to variance in each patient’s preoperative lordosis) (p<0.05). Mean segmental lordosis improved from baseline at six weeks and three, six, and 12 months, and sustained at 24 months by 103.5% (5.0 ± 2.9°), 95.1% (4.6 ± 3.1°), 89.9% (4.4 ± 3.1°), 80.7% (3.9 ± 3.1°), and 77.0% (3.8 ± 2.9°), respectively (p<0.001) (Table 4). The mean anterior disc height significantly increased from baseline by 86.8% (6.8 ± 4.5 mm), 77.5% (6.1 ± 3.0 mm), 70.2% (5.5 ± 3.1 mm), 65.0% (5.1 ± 3.1 mm), and 60.1% (4.7 ± 3.1 mm) at six weeks and three, six, 12, and 24 months postoperatively, respectively (p<0.001). Average middle disc height significantly improved at six weeks and three, six, 12, and 24 months postoperatively by 87.4% (5.8 ± 3.6 mm), 77.3% (5.2 ± 2.8 mm), 71.9% (4.8 ± 2.9 mm), 67.9% (4.5 ± 2.9 mm), and 59.6% (4.0 ± 3.0 mm), respectively (p<0.001). Posterior disc height significantly increased at six weeks and three, six, 12, and 24 months postoperatively by a mean of 77.3% (3.7 ± 2.4 mm), 65.4% (3.1 ± 2.1 mm), 57.3% (2.8 ± 2.1 mm), 49.4% (2.4 ± 2.2 mm), and 44.6% (2.1 ± 2.2 mm), respectively (p<0.001) (Figure 7). Mean neuroforaminal height increased by 41.4% (5.8 ± 3.8 mm), 35.7% (5.0 ± 3.9 mm), 31.2% (4.4 ± 3.7 mm), 26.7% (3.7 ± 3.7 mm), and 21.7% (3.0 ±3.6 mm), respectively (p<0.001) (Table 4, Figure 8). There were 51 patients with abnormal preoperative PI-LL measurements that significantly improved by 9.1 ± 4.9° (p<0.001) and 52 patients with normal preoperative PI-LL measurements that improved by 0.2 ± 4.6° (p=0.748) at 24 months. There was 100% fusion with all operative levels considered radiographically fused via the Brantigan criteria (BSF-3) [17], a 0% pseudoarthrosis rate, and no cases of radiolucency at 24-month follow-up.

**Table 4:** Radiographic parameters

Parameter	Baseline	6 Weeks	3 Months	6 Months	12 months	24 months
Anterior Disc Height (mm)	7.85 (3.3)	14.66 (2.7)*	13.93 (2.2)*	13.36 (2.2)*	12.95 (2.2)*	12.57 (2.0)*
Middle Disc Height (mm)	6.69 (2.9)	12.54 (2.9)*	11.86 (2.4)*	11.50 (2.4)*	11.23 (2.3)*	10.68 (2.3)*
Posterior Disc Height (mm)	4.80 (2.2)	8.51 (1.9)*	7.94 (1.6)*	7.55 (1.6)*	7.17 (1.5)*	6.94 (1.4)*
Neuroforaminal Height (mm)	14.02 (4.0)	19.82 (3.8)*	19.02 (3.9)*	18.39 (3.5)*	17.76 (3.4)*	17.06 (3.3)*
Segmental Lordosis (^○^)	4.87 (3.2)	9.90 (3.7)*	9.50 (3.4)*	9.25 (3.3)*	8.80 (3.0)*	8.62 (2.8)*
Lumbar Lordosis (^○^)	40.35 (8.6)	47.18 (7.0)*	46.17 (6.2)*	45.20 (6.4)*	44.67 (6.7)*	43.43 (6.5)*

Implant-related observations

No implant-related complications were reported, and there was no occurrence of expulsion or implant breakage at any of the operative levels, and no evidence of pseudoarthrosis (at 24 months postoperatively). There was 100% fusion and no secondary surgical procedures were required at the index or adjacent levels reported. There were only one (1/103, 1.0%) case of subsidence and three cases of suspected ASD reported at two years postoperatively. ASD was observed as suspected due to radiographic assessments but was asymptomatic for patients. However, there were no revision surgeries through the 24-month follow-up.

## Discussion

Expandable interbody spacers, with or without adjustable lordosis, have recently been adopted into the orthopedic practice with limited reported clinical data. With any new technology, clinical studies are necessary to determine its impact via post-market surveillance studies. Moreover, evaluation of novel insertion and expansion mechanisms (with various engineering designs) and their effects on clinical outcomes can be more thoroughly assessed through varying, objective, multi-source literature.

DDD with loss of lordosis and disc height is the most common condition for debilitating back pain frequently associated with referred and radicular leg pain [1]. The goal of MIS LLIF for the treatment of DDD, is to restore and maintain disc height and lordosis to correct sagittal alignment with minimal complications associated with ALIF, PLIF, and TLIF procedures [3-6,12-24]. Restoring sagittal alignment is critical to achieving excellent short- and long-term outcomes, as seen in historical literature [15]. Expandable interbody spacers with and without adjustable lordosis allow for insertion at a low profile, as well as expansion in situ to restore disc height and segmental lordosis [16].

The current study showed that the mean VAS pain scores decreased significantly from baseline at 24 months postoperatively by 88% (7.1 ± 1.0 points) and ODI scores significantly decreased by 87.7% (67.4 ± 8.9 points) (p<0.001). These observed clinical outcomes of the current study demonstrate that the use of expandable interbody spacers improves pain and disability scores by two to five times the minimal clinically important difference (MCID) at two-year follow-up [20].

Advantages associated with MIS techniques include less blood loss, low infection rates, and decreased postoperative pain, compared to open approaches [21]. These advantages were similar to the surgical outcomes seen in the current study. A 2016 study by Kim et al. reported no significant intra-and-perioperative complications, dural tears, or infections in their 50 MIS expandable TLIF cases, similar to the MIS expandable LLIF cases in the current study [22]. In contrast, a 2005 retrospective study by McAfee et al. observed intraoperative complications for their 120 spondylolisthesis patients implanted with static TLIF interbody spacers. McAfee et al. also reported seven dural tears (5.8%), two deep wound infections (1.6%), and one (0.8%) case of cage migration. Of these 10 patients, three required revision surgery (2.5% revision rate) [23].

Increases in disc height, neuroforaminal height, lumbar and segmental lordosis were significantly achieved and maintained from preoperative to final follow-up. These significant radiographic improvements demonstrated the efficacy of expandable interbody spacers when used with a lateral lumbar interbody fusion, up to 24-month follow-up. The radiographic and clinical results of the current study compare favorably to findings in the expandable interbody spacer literature. A two-year follow-up study by Boktor et al. included 54 patients and 62 expandable TLIF interbody spacers between December 2013 to December 2015. Boktor et al. also reported low complication rates, short hospital stay, and significant restoration and maintenance of disc height, neuroforaminal height, and segmental lordosis [24]. Boktor et al. found a significant decrease of 37.6% in their patient’s average ODI scores from baseline preoperative values (61.4 ± 17) to two years postoperative (38.3 ± 22.1) (p<0.001) [24]. The current study’s patient population started with a higher mean ODI score of 76.9 ± 7.3 and showed a greater improvement in disability (and a lower standard deviation) of 87.7%, ending with an average score of 9.5 ± 6.3 at 24 months postoperative (a 67.4 ± 8.9 decrease from baseline, (p<0.001)). Boktor et al. reported similar radiographic improvements at 24 months postoperative in their cohort of 37 patients (41 levels): disc height from 8.3 ± 3 to 13.3 ± 2.6, neuroforaminal height from 17 ± 3.4 to 19 ± 2.7(9.6% vs. 21.7% in the current study), segmental lordosis from 5.5 ± 4.3 to 7.3 ± 3.3 (32.7% vs. 77% in the current study), and lumbar lordosis from 40.9 ± 15.7 to 45.4 ± 16 (11% vs. 7.6% in the current study (p<0.001) [24].

A systematic review by Uribe et al. reported a weighted average for segmental lordosis restoration of 3.9° in 23 studies, which compares satisfactorily to the average segmental improvement of 3.8° in the current study [25]. Previous studies [15,26] reported similar improvements in segmental lordosis ranging from 2.5° to 2.8° after LLIF using 10° lordotic cages. A two-year follow-up study by Frisch et al. compared patients treated with lateral expandable interbody spacers to those treated with static interbody spacers, disc height significantly increased by 3.5 mm in the expandable spacer group (p<0.001) [27]. In another study, Sembrano et al. [28] showed a statistically significant correction of disc height and segmental lordosis when comparing lordotic to non-lordotic interbody spacers.

The current study showed minimal complications with implant subsidence observed in only one of the 125 operative levels (0.8%) and no cases of subsequent revision surgery were reported. Other studies have shown similar subsidence results. A systematic review on subsidence after LLIF conducted by Macki et al. [29] reported a 10.3% subsidence rate (n=141/1362 patients, 14 articles) and a reoperation rate for subsidence of 2.7% (n= 41/1470 patients, 16 articles) when various-sized static polymeric spacers were used. Additionally, the current study showed no cases of migration, expulsion, or pseudoarthrosis for MIS LLIF expandable patients. A 2010 study by Aoki et al. observed 125 patients implanted with static TLIF cages and found a 3.2% cage migration rate within three months postoperatively, which they believed to have resulted from undersized cages [30]. Only three cases of ASD were suspected among 103 patients; however, none have required revision surgery thus far in the current study, a longer follow-up is needed to better assess ASD.

Limitations of this study included its lack of a control group and being a single surgeon’s single-site experience. Nevertheless, it provides clinical evidence on the use of lateral expandable interbody spacers for lumbar interbody fusion, which is sparse in the literature. A minimum two-year follow-up is required to assess pseudoarthrosis, which was achieved in this study. This study provides clinical evidence for the sparse, existing literature for expandable lordotic interbody spacers with and without adjustable lordosis for MIS LLIF.

## Conclusions

Correction of segmental lordosis and disc height was achieved in a single-site patient cohort utilizing lateral expandable interbody spacers with and without adjustable lordosis. Significant positive improvements in segmental lordosis, disc height, and indirect decompression were observed at two years postoperative. There was 100% fusion with no revision surgeries reported. Functional outcomes also significantly improved. The use of expandable spacers resulted in postoperative improvements in pain and function at two-year follow-up with few complications.
